# Clinical Implications of a Six-Protein Signature in Bone Metastasis of Renal Cell Carcinoma

**DOI:** 10.7150/jca.88612

**Published:** 2024-04-08

**Authors:** Zheng Liu, Hanwen Mao, Dinggai Chu, Liang Qin, Jiang Wang

**Affiliations:** 1 Department of Oncology, People's Hospital of Dongxihu District, Wuhan, Hubei 430040, P.R. China.; 2 Department of Orthopedic, Tongji Hospital, Tongji Medical College, Huazhong University of Science and Technology, Wuhan, Hubei 430030, P.R. China.

**Keywords:** Bone metastasis, kidney renal clear cell carcinoma, immune check-point inhibitor, clinical omics data cohorts

## Abstract

Bone metastases is prevalent from renal cell carcinoma (RCC) with poor quality of life and prognosis. Our previous proteomics analysis identified dysregulated proteins in the bone-tropism RCC cells. In this study, we further examined the clinical implications of these proteins using multiple clinical cohorts. We identified 6 proteins with significant upregulation in RCC tumor tissue in comparing to tumor adjacent normal tissue (p<0.05). High expression of these 6 protein-encoding genes significantly correlates with a poor survival in the TCGA-KIRC (Kidney renal clear cell carcinoma) cohort (log-rank test p=2.7e-05), and they all individually had a reverse-correlation with the gene expression of VHL and PBRM1 (p<0.001), and positive-correlation with the expression of VEGFA (p<0.001). Further gene set variation analysis (GSVA) revealed positive correlation with Th17 cells enrichment and negative CD8 T cell infiltration in the RCC tumor microenvironment. High expression of these 6 genes in pretreatment tumors favors longer overall survival (OS)(p=0.027) in anti-PDL1 treated patients (n=428). We treated one humeral metastases RCC patient with the anti-PDL1 antibody drug atezolizumab after examined the elevated expression of the 6 proteins in his nephrectomy tumor tissue, the tumor at the fracture site shrunk remarkably after four courses of treatment. These results altogether suggest a clinical implication of the 6-protein signature in RCC bone metastasis prognosis and response to immune-checkpoint inhibitor treatment.

## Introduction

Renal cell carcinoma (RCC), particularly the clear cell subtype, has a high likelihood of distant metastasis, and bone metastasis is observed in approximately 30% of cases among patients with metastatic RCC (mRCC). Historically, the prognosis for mRCC, including those with bone metastasis, has been poor, with a 5-year overall survival (OS) rate of only 12%. However, the introduction of immune checkpoint inhibitors (ICIs) has dramatically improved the 5-year OS rate of mRCC patients to 43% 1. These drugs have now become the standard treatment for mRCC. While there are limited reports, emerging studies have indicated an improvement in the median survival time of mRCC patients with bone metastases by ICIs treatment 2, however, further research and clinical evidence are still needed to fully understand their therapeutic effects in this context.

It is observed that RCC bone metastasis exhibits early onset and highly osteolytic features. The development of bone metastasis from RCC often occurs before diagnosis and may be accompanied by metastases in other organs within a relatively short period. The osteolytic changes associated with RCC bone metastases can lead to skeletal-related events (SRE) that have a significant impact on patients' quality of life and prognosis. Our previous research involved a comprehensive proteome analysis comparing bone-seeking RCC cells with their parental cells, leading to the identification of 26 significantly up-regulated proteins in the bone-seeking RCC cells 3. These proteins were hypothesized to be potential driving factors for bone metastatic colonization of RCC. Specifically, we validated the function of the protein STIP1 in promoting RCC tumor cell proliferation and migration/invasion through the autocrine STIP1-ALK2-SMAD1/5 pathway; and enhancing osteoclast differentiation and proliferation through the paracrine STIP1-PrPc-ERK1/2 pathway 4.

In this study, we aimed to examine the clinical implications of these proteins using multiple clinical cohorts, including the TCGA Kidney renal clear cell carcinoma (KIRC) RNAseq dataset (n=613), a combined RCC microarray dataset (n=556) and a combined transcriptomic dataset of cancer patients treated with ICIs (n=1,361). Analysis using these cohorts identified a 6-protein signature associated with bone metastasis in RCC, including HPCAL1, SLC9A3R1, HSP90B1, EZR, RCN1, and CHMP2A. Since the interaction between tumor cells and stromal cells, including immune cells, is largely mediated by proteins, we hypothesized that these bone metastatic driving proteins may be associated with specific immune landscapes that could potentially indicate treatment response to checkpoint inhibitors. The analysis of clinical omics data cohorts and examination of the relationship between bone metastatic-associated proteins and immune landscape could provide valuable insights into the potential use of these proteins as prognostic or predictive markers, particularly in the context of immune checkpoint inhibitor therapy.

## Materials and Methods

### Clinical cohorts

The TCGA-KIRC RNAseq dataset was used to examine the mRNA expression of the 26 protein-encoding genes in paired tumor vs tumor-adjacent normal tissue (n=72) and non-paired tumor vs normal tissue (n=541 vs 72) by the Wilcoxon rank sum test. Samples with survival information in this dataset (n=415) was used for log-rank Kaplan-Meier analysis of the 26 genes' expression level in association to patient overall survival time, using the median expression of the gene set as the cutoff. A combined RCC microarray dataset (n=556) 5 was used to examine the Spearman correlation between the 26 genes and commonly mutated RCC genes, i.e., tumor suppressor gene von Hippel-Lindau (VHL), and tumor-promoting genes vascular endothelial growth factor A (VEGFA), and Polybromo 1 (PBRM1). Enrichment of immune cell infiltration in correlation with gene expression was conducted using the single-sample gene set enrichment analysis (ssGSEA) in the R-GSVA [1.46.0] package 6, with the immune cell markers published by Bindea, et al. 7. A combined transcriptomic dataset of cancer patients treated with anti-PD1 and anti-PDL1 immune-checkpoint inhibitors (n=1,361) 8 was used to explore the risk of overall survival (OS) and progression-free survival (PFS) in pretreatment tumor samples in association with the gene expression levels. The Human Protein Atlas database 9 (https://www.proteinatlas.org/) contains 26 tissue cores from 13 KIRC patients was used to confirm the protein expression levels of the bone metastasis-related proteins.

### Patient tumor samples and immunochemistry

Twenty-five tumor tissue samples were used to examine the expressions of the bone metastasis-associated proteins, including 12 pairs of primary RCC tumor tissue from nephrectomy and bone metastasis tissues from biopsy that were retrospectively collected from tissue bank of Tongji Hospital, and one primary RCC sample from the one case received anti-PDL1 treatment as described below. The clinical characteristics of the 12 RCC patients are provided in **Table S1**. Inclusion criteria includes: patients with bone metastatic RCC histologically or cytologically documented; patients completed systemic therapy for advanced disease for at least 30 days. Patients with previous or concurrent malignancy, diabetes, or major medical illnesses were excluded. The diagnosis of primary RCC as clear cell histotype and bone metastasis was done according to the WHO classification criteria by the Department Pathology of Tongji Hospital. The study was approved by the IRB committee of Tongji Medical College, Huazhong University of Science and Technology and was carried out in accordance with the Declaration of Helsinki. Consent forms from all participants were obtained.

The formalin-fixed, paraffin-embedded tissue sections were stained with the primary and secondary antibodies against the 6 proteins (**Table S2**) and detected by the Ventana Basic DAB (3,3-diaminobenzidine) Detection kit (Boster Biotechnology, Wuhan, China). Slides were evaluated independently by two pathologists who were blinded to the clinicopathological data and the patients' identities. The overall immunohistochemical score (histoscore) was calculated as the percentage of positive tumor cells (0-100%) multiplied by staining intensity (0 = negative, 1 = weak, 2 = moderate, 3 = strong). Therefore, the total histoscore ranged from 0 to 300.

### Case presentation

A 52-year-old man, who was previously diagnosed with clear cell RCC and underwent a left nephrectomy, experienced a sudden right upper arm pain nine years after the surgery. X-ray radiographs revealed a fracture in the diaphysis of the humerus with osteolytic changes. Further investigation through a tumor biopsy confirmed that the bone metastasis was originating from RCC. We examined strong immune-reactive staining of the 6 bone metastasis-associated proteins in his primary RCC tumor tissue, and ICI treatment using atezolizumab, which is an anti-PDL1 antibody drug, was immediately initiated. Atezolizumab was administered as monotherapy to the patient. Additionally, to address the pathological fracture, functional brace fixation was employed for treatment. Following four cycles of atezolizumab treatment, significant shrinkage of the tumor at the pathological fracture site was observed, and no immune-related adverse events were reported.

## Results

### Six bone metastasis-associated proteins significantly upregulated in primary RCC tumors

**Figure 1:**
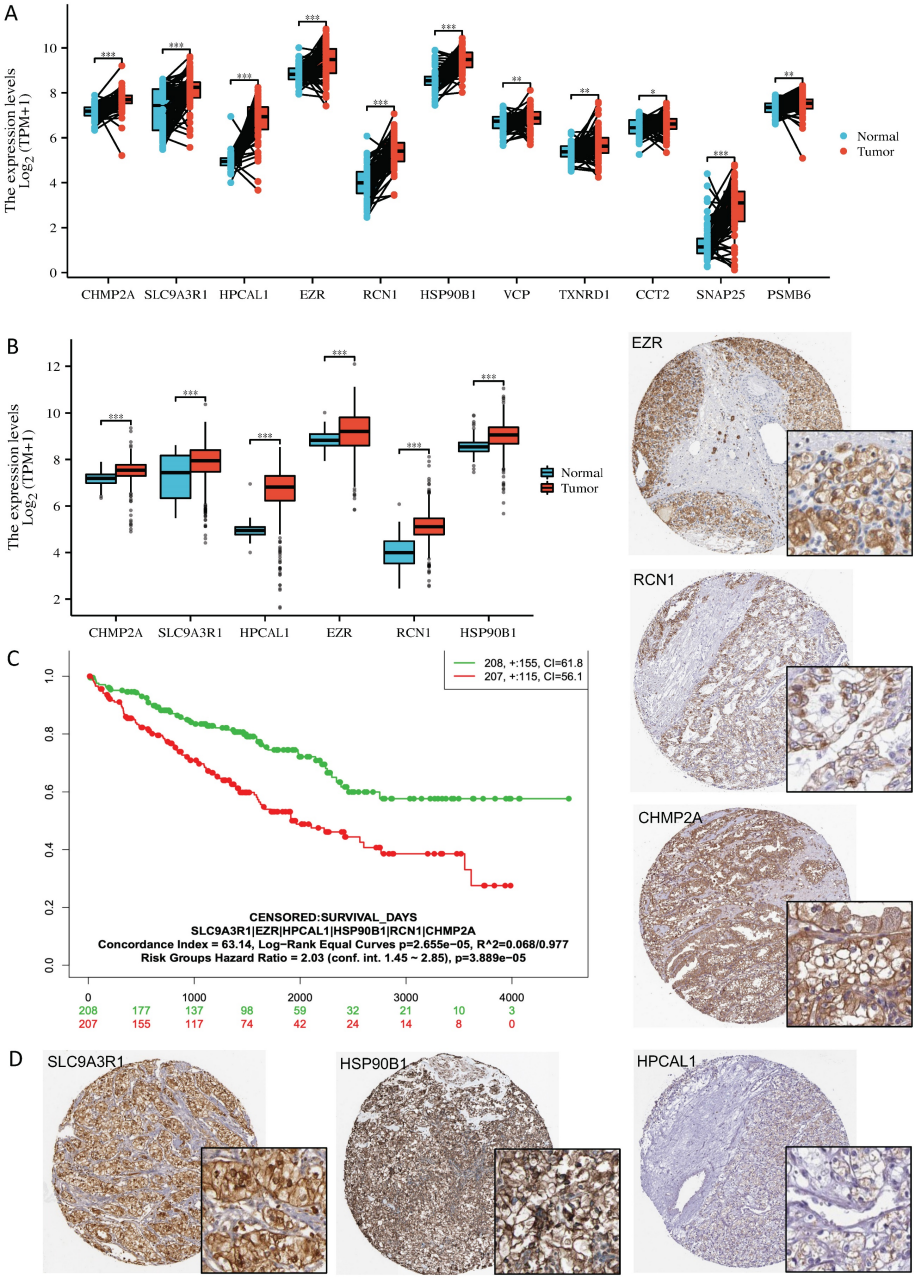
** Six bone metastasis-associated proteins significantly upregulated in primary RCC tumors. A.** Eleven of the 26 protein encoding genes are significantly up-regulated in paired tumor vs tumor-adjacent normal tissue (n=72) in the TCGA-KIRC RNAseq dataset. Wilcoxon rank sum test, *** p<0.001; *p<0.05. **B.** Six of the 11 protein encoding genes are up-regulated in a larger cohort of tumor samples vs normal tissue (n=541 vs 72) in the TCGA-KIRC RNAseq dataset. Wilcoxon rank sum test, *** p<0.001; *p<0.05. **C.** Samples with survival information in the TCGA-KIRC RNAseq dataset (n=415) was used for log-rank Kaplan-Meier analysis of the 6 genes' expression level in association to patient overall survival time, using the median of mean expression of the gene set as the cutoff. Log-rank test, p=2.66e-05. **D.** Expression of the 6 proteins in KIRC tumor tissue cores in The Human Protein Atlas database 9 (https://www.proteinatlas.org/).

Our previous studies using proteomics analysis discovered 26 significantly up-regulated proteins in the bone-seeking RCC cells in comparing to non-bone-seeking RCC cells 3. These proteins may have a potential in driving bone metastasis development in RCC. To explore the relevance of these proteins in primary RCC, we first examined the transcriptomic expression of these proteins in the tumor and normal tissue matched samples in the TCGA-KIRC dataset (n=72). Eleven of the 26 genes showed significant upregulation in the tumor tissue (p<0.05) (**Fig. 1A**, **Table S3**). Using the larger sample size of total 541 tumor samples vs 72 normal tissue in the TCGA-KIRC dataset, we further identified 6 of the 11 genes showed remarkable and consistent upregulation in the tumor samples (p<0.001) (**Fig. 1B, Table S3**). High expression (median as the cutoff) of the 6 genes as a set correlated with a worse overall survival in the TCGA-KIRC cohort (log-rank test, p=2.7e-05) (**Fig. C**). The Human Protein Atlas database confirmed the medium to high expression of the 6 proteins, i.e., SLC9A3R1, HSP90B1, EZR, RCN1, CHMP2A and HPCAL1 in the 26 tissue cores from 13 KIRC patients (**Fig. 1D**). These results clearly indicate the clinical relevance of the 6 proteins in RCC.

### Six bone metastasis-associated proteins significantly upregulated in RCC bone metastases

**Figure 2:**
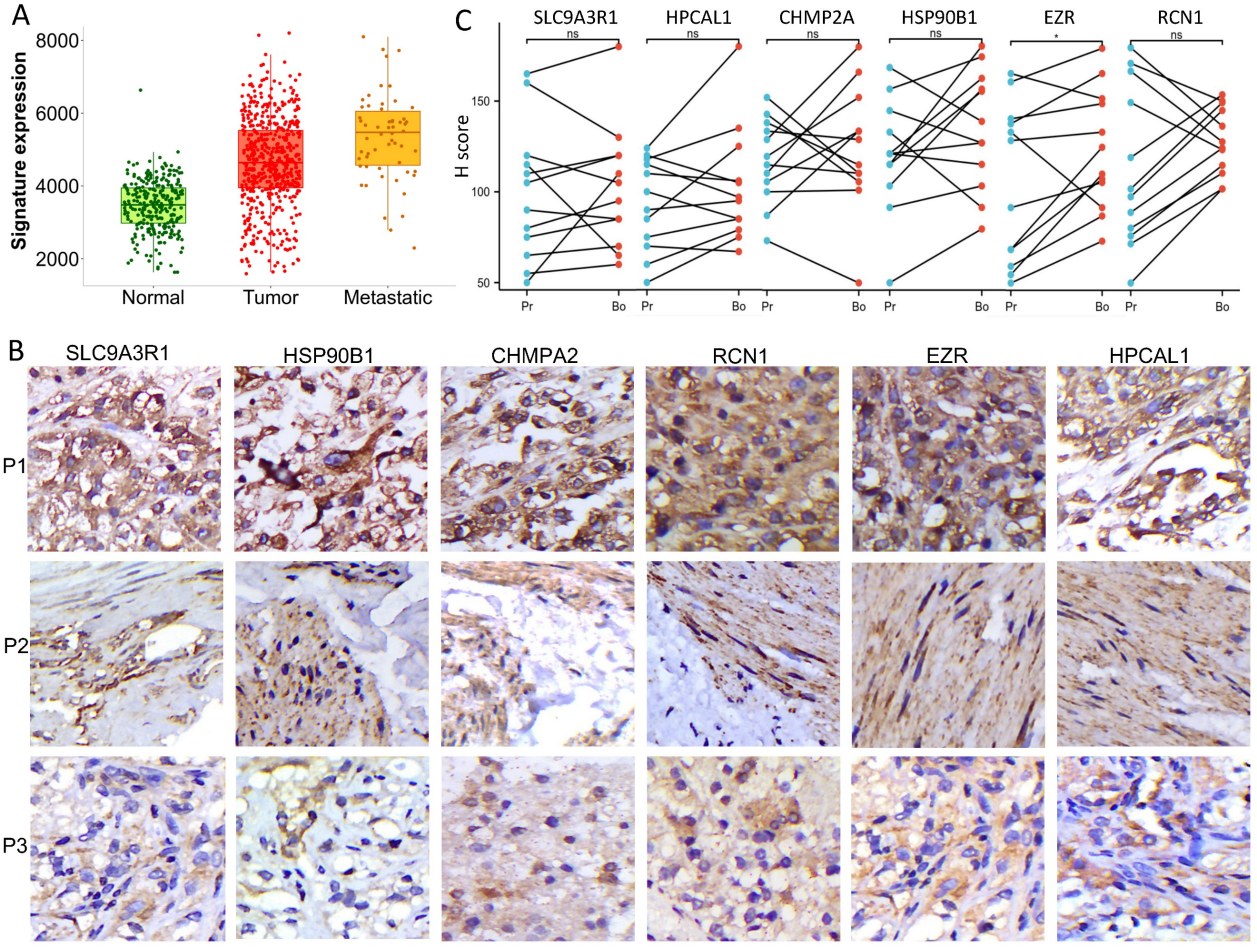
** Six bone metastasis-associated proteins significantly upregulated in RCC bone metastases. A.** Gene expression of the 6 proteins showed significant increase in mRCC vs RCC (fold change = 1.18) and RCC vs normal tissue (fold change =1.34) in a combined RCC microarray dataset containing 277 normal kidney tissue, 556 RCC tumors and 58 mRCC tumors 5. Kruskal-Wallis test, p= 6.7 e-52. **B.** Representative immunochemistry staining images of the 6 proteins in the bone metastasis samples of 3 individual patients. **C**. Protein expression (H score) of the 6 proteins in the 12 paired samples. Pr-primary tumor, Bo-bone metastasis; *, p<0.05, ns, non-significant, Wilcoxon signed rank test.

To our knowledge, no publicly available RCC clinical cohort contains bone metastasis annotation. By mining a combined RCC microarray dataset which contains 277 normal kidney tissue, 556 RCC tumors and 58 mRCC tumors 5, expression of the 6 genes as a signature showed dramatic increase in mRCC vs RCC (fold change = 1.18) and RCC vs normal tissue (fold change =1.34) (Kruskal-Wallis p= 6.7 e-52) (**Fig. 2A**). We further verified the relevance of the 6 proteins in RCC bone metastasis using in-house collected patient tissues. We performed immunochemistry staining and quantified the expression of the 6 proteins in 12 matched primary RCC and bone metastasis samples (n =12). Medium to strong immuno-reactivities of all the 6 proteins were observed on the cell membrane or in cytoplasm of bone metastasis tumor cells (**Fig. 2B**); they also expressed fairly in the primary RCC tumor cells, there is no obvious correlation of their expression level between the primary tumors and bone metastatic tumors (**Fig. 2C**).

### Correlation of the 6 protein-encoding genes with RCC feature genes and immune cell enrichment

**Figure 3:**
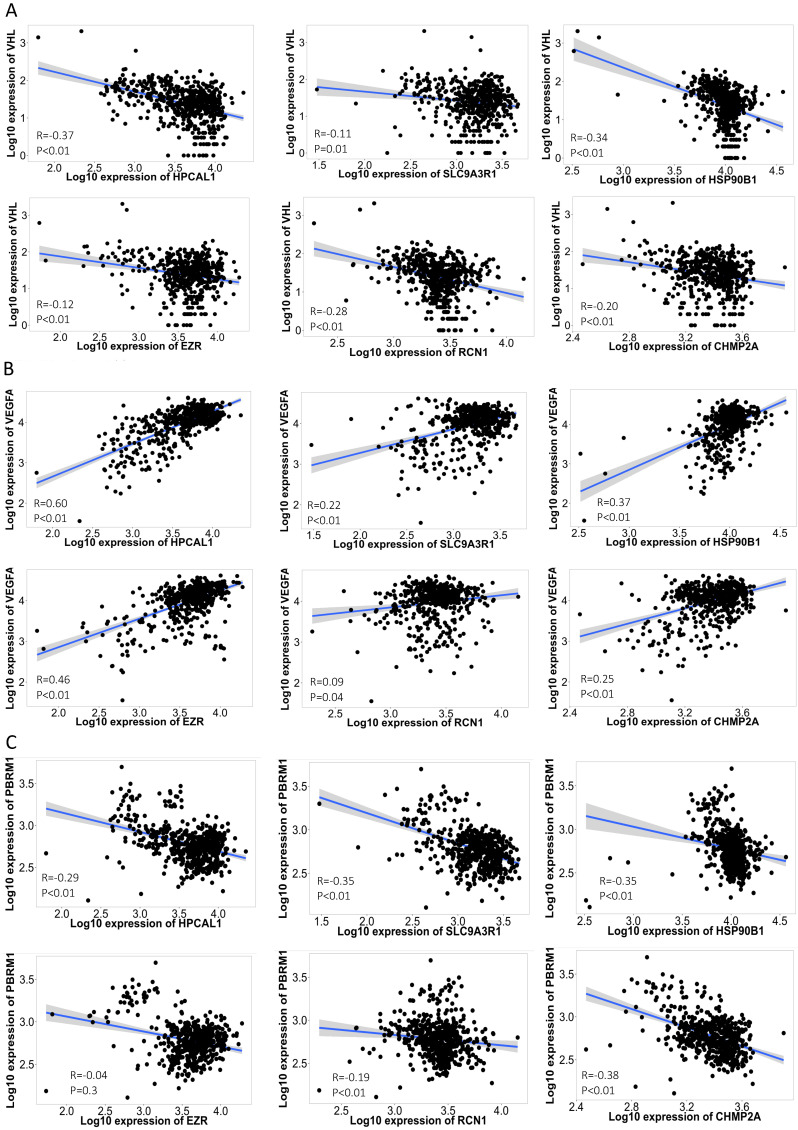
** Correlation of the 6 protein-encoding genes with RCC feature genes.** Each of the 6 protein-encoding genes showed a negative-correlation with VHL expression (A), and positive-correlation with VEGFA expression (B) in the 556 RCC cohort 5. Negative-correlation with PBRM1 expression was also identified in 5 of the 6 genes in the same 556 RCC cohort (C). Spearman correlation co-efficiency and p value is listed in each figure.

**Figure 4:**
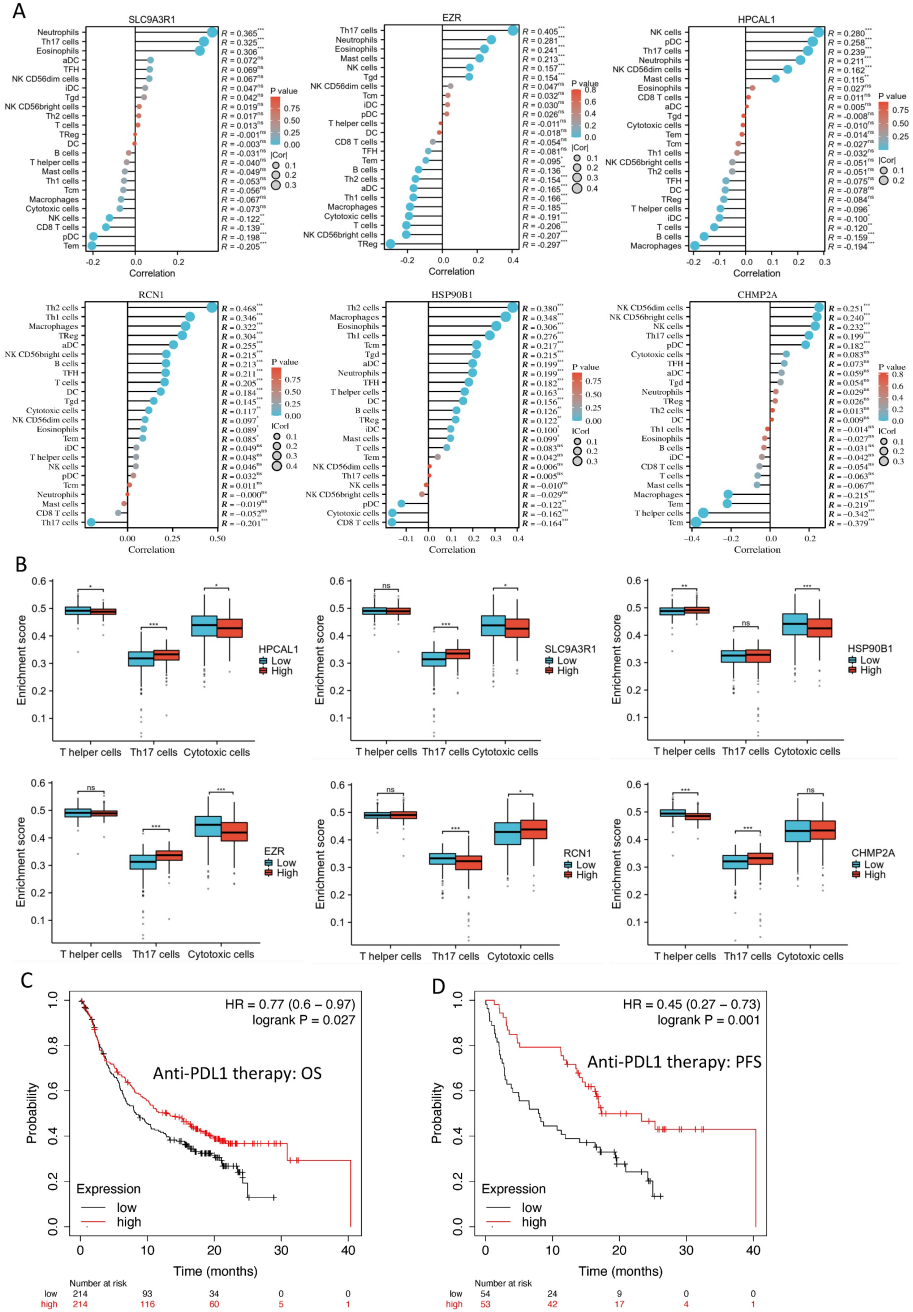
**Correlation of the 6 protein-encoding genes with RCC immune cell enrichment. A.** ssGSEA analysis of the 6 protein-encoding genes in association to 24 types of immune cells in RCC tumors. In the TCGA-KIRC cohort. **B.** Th17, T helper and cytotoxic T cell enrichment in the high vs low expression of the 6 genes. Two group t-test, * p<0.05, ** p<0.01, *** p<0.001. **C-D.** Kaplan-Meier analysis of the 6 genes' expression level in association to patient OS (**C**) and PFS (**D**), using the median of mean expression of the gene set as the cutoff, in a combined cohort of 428 or 107 patients that treated with either atezolizumab or durvalumab anti-PDL1 immunotherapy 8.

The disruption of the VHL tumor suppressor gene and overexpression of VEGFA are considered the hallmark of clear cell RCC. Each of the 6 protein-encoding genes showed a reverse-correlation with VHL expression (p<0.01), and positive-correlation with VEGFA expression (p<0.01) in the RCC cohort (n=556) (**Fig. 3A-B**). PBRM1 is another frequently altered tumor suppressor gene in RCC, and loss of PBRM1 promotes response to immunotherapy in RCC 10. Negative-correlation with PBRM1 expression was also identified in 5 of the 6 protein-encoding genes in the same RCC cohort (n=556) (p<0.01) (**Fig. 3C**). To further explore the relevance of these 6 genes in tumor immune microenvironment, we performed ssGSEA analysis using the TCGA-KIRC cohort. Among the 24 types of immune cells (**Fig. 4A**), Th17 cells enrichment is mostly dominant and positively correlated with high expression of 4 genes (p<0.01) (**Fig. 4B**). Overall, high expression of the 6 genes is associated with an immunosuppressive environment (**Fig. S1**), as demonstrated by negative correlation with CD8 cytotoxic T cell and CD4 help T cell infiltration in the RCC tumors (**Fig. 4B**). We further identified that high expression of these 6 genes in pretreatment RCC tumors associated with a favorable prognosis in a combined cohort of 428 patients that treated with either atezolizumab or durvalumab anti-PDL1 immunotherapy 8 (**Fig. 4C**). Patients with high expression of the 6 genes in the tumors had a median 12.85 months OS vs the 8.25 months in the low expression group (log-rank test p=0.027). Similar favorable PFS was observed in 107 patients with PFS information (log-rank test p=0.001) (**Fig. 4D**).

### A bone metastatic patient with positivity of the 6 proteins in the RCC tumor responded well to anti-PDL1 treatment

**Figure 5:**
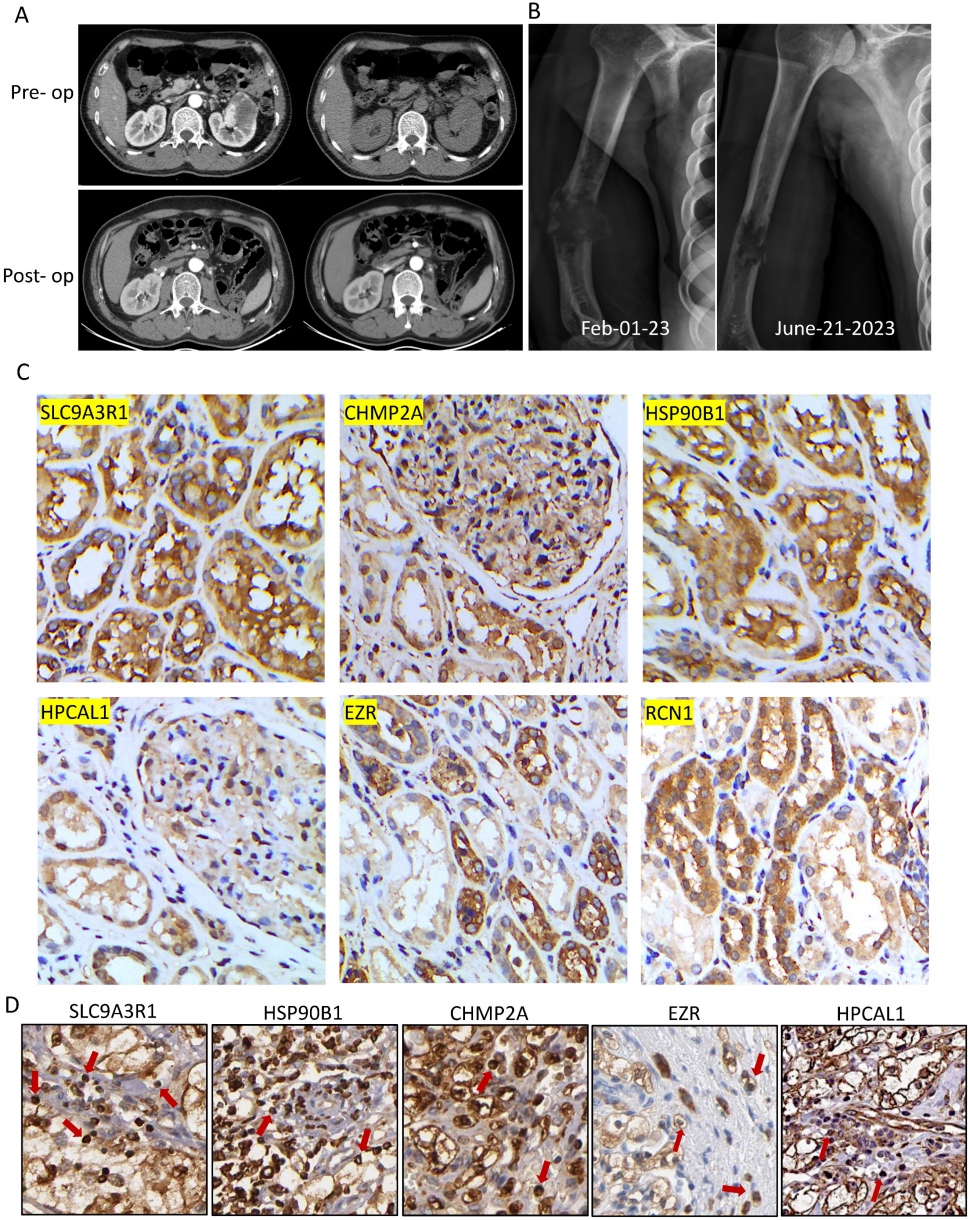
** A bone metastatic patient with positivity of the 6 proteins in the RCC tumor responded well to anti-PDL1 treatment. A.** Magnetic resonance imaging (MRI) showed left kidney tumor mass before and after surgical removal (left: T1-weighted image, right: fat-suppressed T2-weighted image). **B.** Nine years later, a right humerus fracture with osteolytic change was revealed by X-ray, and it was confirmed by biopsy as a pathological fracture due to bone metastasis. Following four cycles of atezolizumab treatment, significant shrinkage of the tumor at the pathological fracture site was observed. **C-D.** Positivity of the immunochemistry staining of 6 proteins were examined not only in the primary RCC tumor cells in the treated patient but also infiltrating T cells (red arrows) in the Human Protein Atlas.

This patient with nephrectomy of clear cell RCC developed humerus metastasis nine years later (**Fig. 5A-B**). In comparing to the protein expressions in the 12 RCC tumors, this patient had strong immune-reactive expression of the 6 bone metastasis-associated proteins in his primary RCC tumor tissue (**Fig. 5C**). We further detected medium to high expression of 5 out of 6 proteins on the infiltrating T cells in the primary tumor (**Fig. 5D**). Following four cycles of anti-PDL1 drug atezolizumab treatment in March 2023, significant shrinkage of the tumor at the pathological fracture site was observed, and no immune-related adverse events were reported. The patient reported great improvement on his shoulder joint function and quality-of-life and continued to be followed up with radiological examinations every six months.

## Discussion

Bone metastasis is a poor prognostic factor in RCC 11, 12. Our study identified a 6-protein signature associated with bone metastasis in RCC and suggests its potential as a predictive biomarker for anti-PDL1 ICI treatment in bone metastasis. The proteins identified in the signature are HPCAL1, SLC9A3R1, HSP90B1, EZR, RCN1, and CHMP2A. The evidence supporting this protein signature was derived from multiple independent clinical cohorts, as well as in-house collected patient tumor samples. The findings were further validated through consistent protein and transcription expression changes observed in clinical cohorts. It is noted that while ICIs have become the standard treatment for metastatic RCC, their therapeutic effect on bone metastases is not extensively studied, and there remains a challenge in optimizing treatment selection based on predictive markers for individual patients.

We reported a case involves a patient with bone metastatic RCC who was treated with an anti-PDL1 ICI based on the positivity of the 6-protein signature in the primary RCC tumor. Radiological examination confirmed a significant tumor shrinkage at the pathological fracture site after four cycles of treatment. This study serves as an initial step towards rigorously evaluating the 6-protein signature in the context of ICI treatment for RCC bone metastasis. Further research and clinical trials are needed to validate the findings, assess the predictive value of this protein signature, and determine its utility in guiding treatment decisions for patients with RCC and bone metastasis.

The 6 proteins were screened out from intracellular proteomics analysis of bone-seeking RCC cells 3, and their relevance to kidney cancers has been rarely reported. HPCAL1 is a calcium-binding protein that has recently been identified as a specific autophagy receptor 13. Autophagy is a cellular process involved in recycling and degradation of cellular components and has been implicated in various aspects of cancer, including metastasis. SLC9A3R1 is a solute carrier protein that regulates protein trafficking. The SLC9A3R1-related signaling pathway has been shown to activate autophagy processes 14. HSP90B1 is a molecular chaperone that plays a role in the processing and transport of secreted proteins. It is involved in protein folding and stabilization. EZR is a cytoplasmic peripheral membrane protein that participates in various biological processes, including cell adhesion, migration, cytokinesis, and formation of surface structures. EZR activation has been reported to mediate hypoxia-induced autophagy in tumor cell self-renewal and colon cancer progression 15. RCN1 is a Ca^2+^‐binding protein locates at both cell membrane and endoplasmic reticulum (ER). It has been shown to inhibit ER stress-induced apoptosis 16. CHMP2A is an autophagy-related protein 17 that has recently been reported to regulate tumor sensitivity to natural killer cell-mediated cytotoxicity 18. Based on the prior knowledge, there seems to be a common theme of autophagy activation associated with several of these proteins in RCC bone metastasis. Autophagy has been studied extensively in cancer metastasis, as it can modulate tumor cell motility, invasion, cancer stem cell behavior, resistance to cell death (anoikis), epithelial-to-mesenchymal transition, tumor cell dormancy, and immune evasion 19. However, it is important to note that while these proteins show potential biological relevance to RCC bone metastasis based on their functions and prior knowledge, further research and experimentation are necessary to understand the precise mechanisms and functional significance of these proteins in the context of RCC bone metastasis.

High expression of the 6 proteins was associated with an immunosuppressive environment in RCC, as evidenced by lower ImmuneScore and ESTIMATEScore 20 (**Fig. S1**). This suggests that these proteins may play a role in modulating the immune response in RCC. Specifically, high expression of the 6 genes was negatively correlated with infiltrations of CD8 cytotoxic T cells and CD4 helper T cells. This is consistent with previous reports indicating that clear cell RCC is characterized by an immunosuppressive environment that hinders the function of immune cells 21. The positive association of the 6 proteins with Th17 cells and neutrophils suggests a potential role in promoting osteoclastogenesis and osteolytic features in RCC bone metastasis. Th17 cells are known to produce pro-osteoclastic cytokines 22, IL-17 produced by Th17 cells can recruit neutrophils into tumor microenvironment to activate tumor cells in expressing metastasis-related genes 23. Interestingly, the higher expression of the 6 genes in pretreatment RCC tumors correlated with a favorable prognosis in patients treated with anti-PDL1 immunotherapy. However, the expression of PDL1 was not detected in all the in-house RCC samples, and PD1 was lowly expressed in a subset of the tumor samples (**Fig. S2**). On the other hand, 5 of the 6 proteins were found to be highly expressed on infiltrating T cells in the RCC tumor of the bone metastatic patient. Coincidently, the single cell RNA data in the Human Protein Atlas database also suggests expression of these 5 genes in T cell cluster in kidney tissue (**Fig. S3**). This suggests a potential association between the 6 proteins and the immune checkpoint molecules PD1 or PDL1. Although more experimental data are needed to support the connections between the 6 proteins and PD1 or PDL1, it is hypothesized that these proteins, involved in protein trafficking, folding, and stabilization, could regulate the functional expression of PD1 or PDL1 in RCC. Overall, our study provides valuable insights into the relationship between the 6 proteins, the immune environment in RCC, and the potential implications for immunotherapy response. Further experimental investigations are necessary to validate and expand upon these findings, particularly regarding the interactions between the 6 proteins and immune checkpoint molecules in RCC.

A few case reports 24, 25, along with our own case, highlight the significant improvement of bone metastases from RCC with ICI treatment. This is consistent with the growing evidence suggesting the efficacy of ICIs in treating mRCC. In our study, the rationale for administering anti-PDL1 treatment to the patient was based on the 6-protein positivity observed in the RCC tumor. The 6-protein signature correlates with a good prognosis in the anti-PDL1 treatment cohort, but not in the anti-PD1 treatment cohort (**Fig. S4**). This discrepancy might be attributed to the enrichment of PDL1 expression in tumor cells in bulk RNAseq analysis, while PD1 is primarily expressed on T-cell membranes. As T cells were not enriched in the tumor RNAseq analysis, the correlation with the 6-protein signature may not be evident in the anti-PD1 cohort. It is unfortunate that the patient's refusal for surgery prevented the examination of the 6 proteins in the bone metastatic tissue. Understanding the immune landscape in bone metastasis is crucial, as the bone marrow consists of a diverse array of immune cells that can have both pro-tumor and anti-tumor effects within the bone metastatic niche 26. Further systemic investigations are needed to elucidate the interplay between the immune niches and the 6 proteins in RCC bone metastasis.

We are aware of the limitations of the current study, first, the functional significance of the 6 proteins was not investigated in experimental models of RCC bone metastasis. Second, the regulatory mechanism between the 6 proteins and immune checkpoint molecules need validation. Third, we only reported one clinical case, further clinical trials are needed to validate the efficacy of ICIs in treatment of RCC bone metastasis.

## Conclusions

Our study identified a 6-protein signature associated with bone metastasis in RCC and suggests its potential as a predictive biomarker for anti-PDL1 ICI treatment in bone metastasis. Overall, our study contributes to the growing body of evidence supporting the use of ICIs in treating RCC bone metastases. The correlation between the 6-protein signature and treatment response underscores the potential of these protein sets as a predictive biomarker. Further research is needed to explore the immune landscape of bone metastasis and the specific roles of the 6 proteins in this context.

## Supplementary Material

Supplementary figures and tables.
