# Supplementary material for: Clinical Implications of a Six-Protein Signature in Bone Metastasis of Renal Cell Carcinoma
Source: J Cancer. 2024 Apr 8;15(10):3034–44. doi: 10.7150/jca.88612 (PMC11064255; doi:10.7150/jca.88612)
Supplement: Supplementary file 1 — Supplementary figures and tables. [file jcav15p3034s1.pdf]

Table S1. Clinical and pathologic features of the RCC cohort

ID	Age at surgery	Gender	Differentiation stage	Pathologic T	N stage	Tumor size (Max Dimension)	Nephrectomy	Biopsy location of BM
C1	59	Female	G2	pT2b	N0	101 mm	radical, right	Sacrum
C2	62	Male	G3	pT1b	N1	65 mm	radical, left	Humerus
C3	60	Male	G2	pT1a	N1	28 mm	radical, left	Spinal column
C4	61	Female	G2	pT3a	N1	75 mm	radical, left	Spinal column
C5	68	Male	G3	pT2b	N2	60 mm	partial, right	Sacrum
C6	58	Female	G1	pT3a	N1	98 mm	radical, right	Humerus
C7	62	Male	G2	pT3a	N0	84 mm	radical, right	Sacrum
C8	57	Male	G3	pT2a	N1	85 mm	radical, right	Spinal column
C9	48	Female	G2	pT2b	N2	62 mm	radical, left	Sacrum
C10	68	Female	G2	pT1b	N2	55 mm	radical, left	Humerus
C11	53	Male	G3	pT3b	N1	58 mm	radical, right	Spinal column
C12	60	Male	G3	pT2a	N1	72 mm	radical, left	Sacrum

Table S2. Antibody list

Antibody	vendor	Cat #
SLC9A3R1	Invitrogen	PA1-090
HPCAL1	Sigma Aldrich	MABC1138
HSP90B1	Thermal Fisher Scientific	36-2600
CHMP2A	Invitrogen	PA5-59454
EZR	Abcam	ab40839
RCN1	Abcam	ab205927
PDL1	Cell Signaling	13684
Goat anti rabbit IgG HRP secondary antibody	Invitrogen	31460

Table S3. Statistical results for TCGA-KIRC expression of the genes in 72 paired tumor/tumor adjacent normal tissue (A) and non-paired 541 tumor vs 72 normal tissue (B).

A

GENE	Group I	Group J	statistic	difference (J-I)	CI(95% CI)	p value
VCP	Normal	Tumor	848	0.18242	0.045309 - 0.3063	0.0090
SLC9A3R1	Normal	Tumor	414	0.85756	0.56003 - 1.1423	4.47e-07
HSP90B1	Normal	Tumor	83	0.91302	0.74232 - 1.0684	5.01e-12
EZR	Normal	Tumor	460	0.57385	0.37136 - 0.77725	1.67e-06
TXNRD1	Normal	Tumor	766	0.24455	0.09573 - 0.37493	0.0021
CCT2	Normal	Tumor	934	0.14428	0.012668 - 0.28793	0.0332
RCN1	Normal	Tumor	22	1.3586	1.1705 - 1.5656	4.25e-13
SNAP25	Normal	Tumor	180	1.6599	1.3043 - 1.951	2.01e-10
CHMP2A	Normal	Tumor	162	0.56187	0.47516 - 0.64279	1.03e-10
PSMB6	Normal	Tumor	745	0.17701	0.074163 - 0.27075	0.0014
HPCAL1	Normal	Tumor	50	1.952	1.7399 - 2.1261	1.34e-12

B

GENE	Group I	Group J	statistic	difference (J-I)	CI(95% CI)	p value
SLC9A3R1	Normal	Tumor	1.312e+04	0.5199	0.27332 - 0.78594	6.76e-06
HSP90B1	Normal	Tumor	1.017e+04	0.49668	0.37173 - 0.62695	4.42e-11
EZR	Normal	Tumor	1.414e+04	0.3622	0.17508 - 0.54017	0.0002
RCN1	Normal	Tumor	4779	1.1333	0.96082 - 1.3003	2.23e-25
SNAP25	Normal	Tumor	6735	1.647	1.3704 - 1.903	1.8e-19
CHMP2A	Normal	Tumor	8322	0.377	0.29247 - 0.46192	2.78e-15
HPCAL1	Normal	Tumor	2866	1.8668	1.7301 - 2.0037	5.9e-32

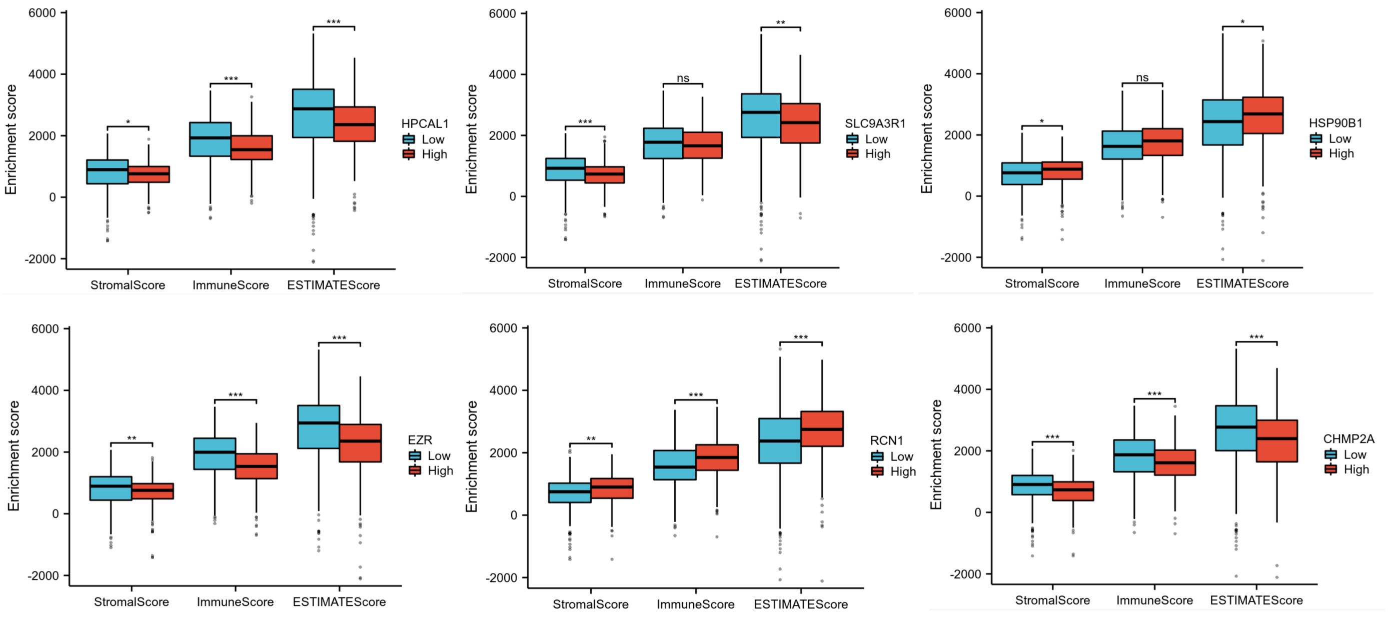

Fig S1. Expression of the 6 genes in association to StromaScore, ImmuneScore and ESTIMATEScore in the TCGA-KIRC cohort.

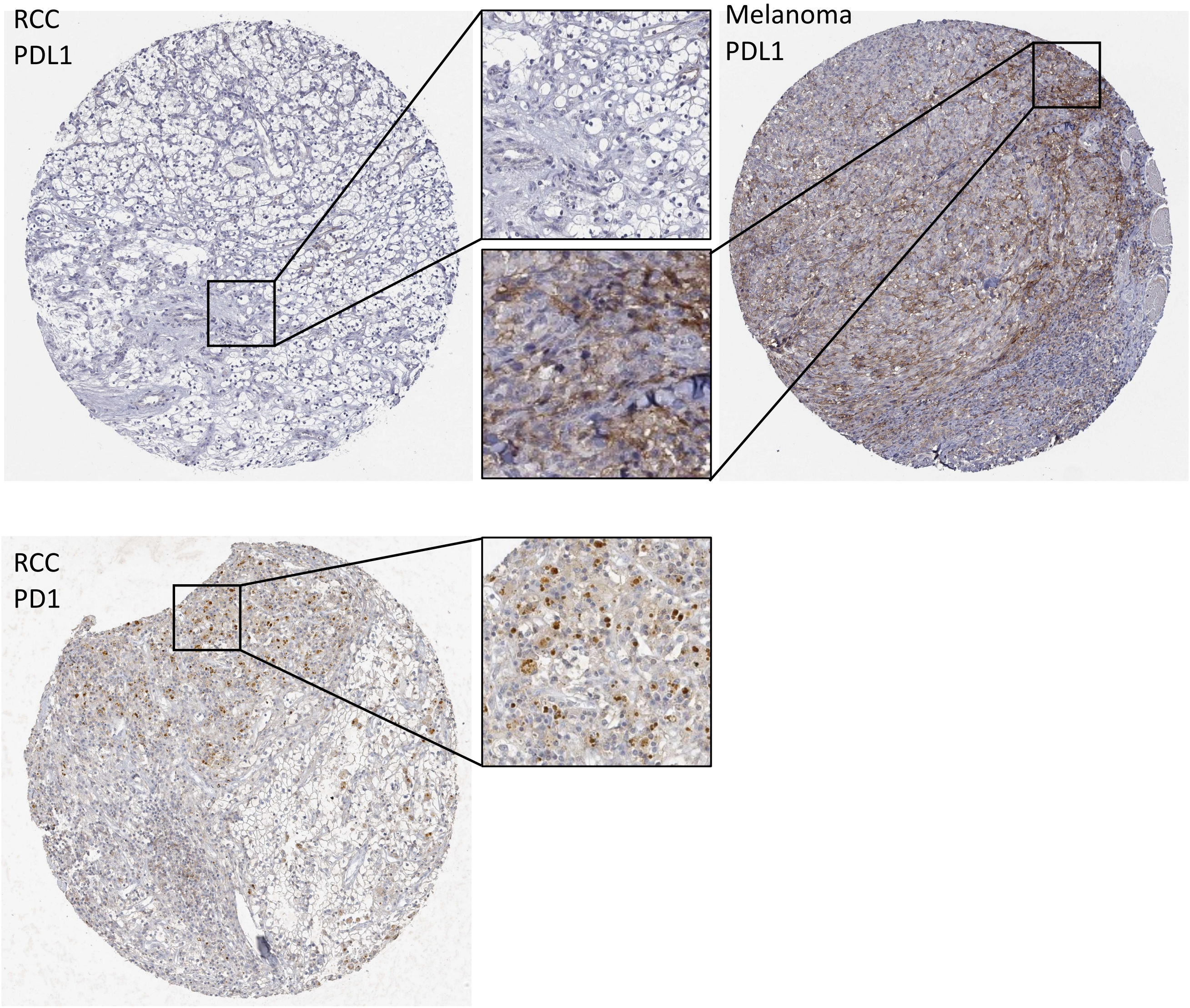

Fig S2. Expression of PDL1 and PD1 in the RCC tumor samples in the Human Protein Atlas. PDL1 was not detected in the RCC tumor while the antibody stained positive in a melanoma tumor sample.

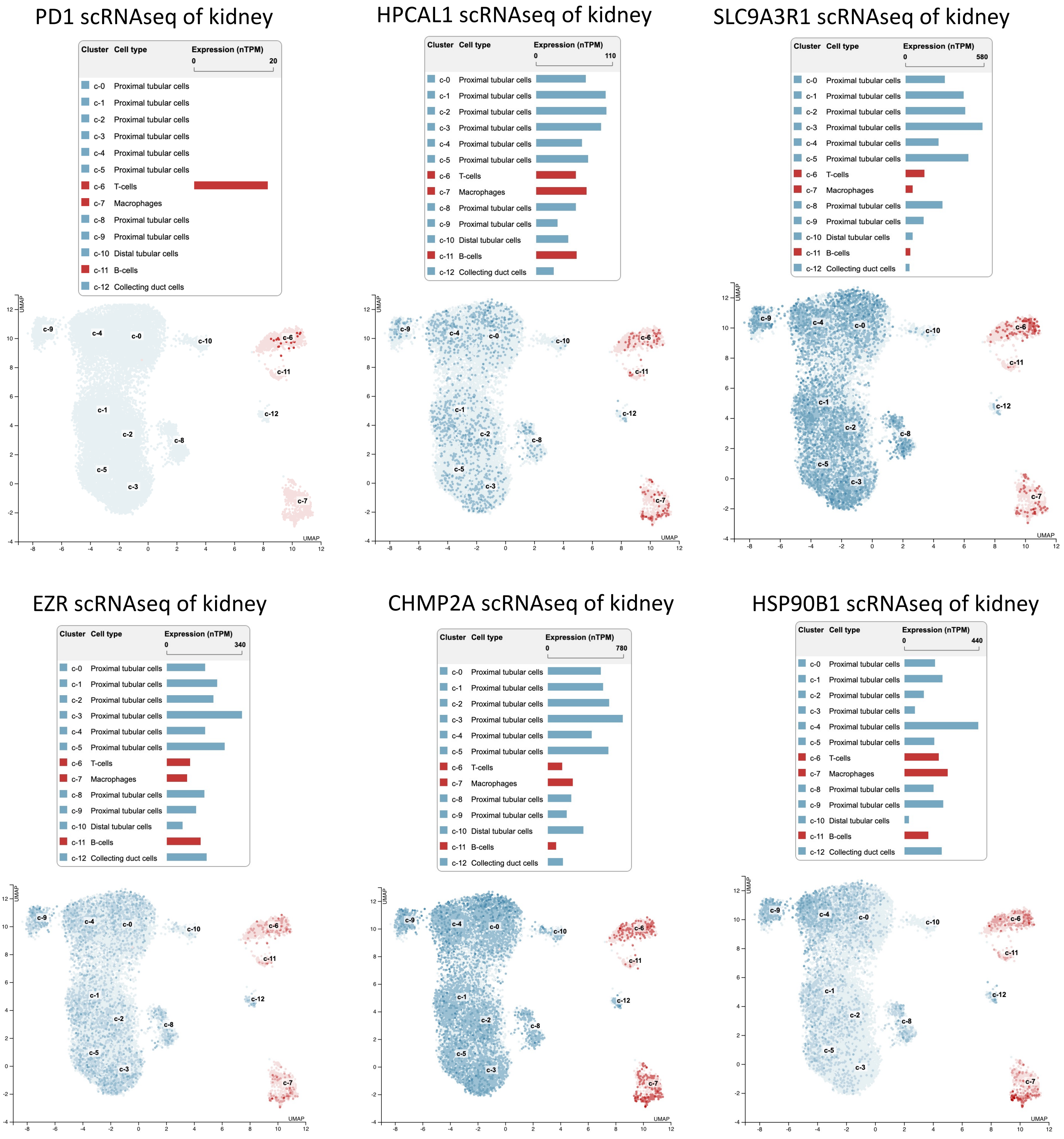

Fig S3. Single cell RNAseq data in the Human Protein Atlas showing expression of the 6 genes in kidney tissue.

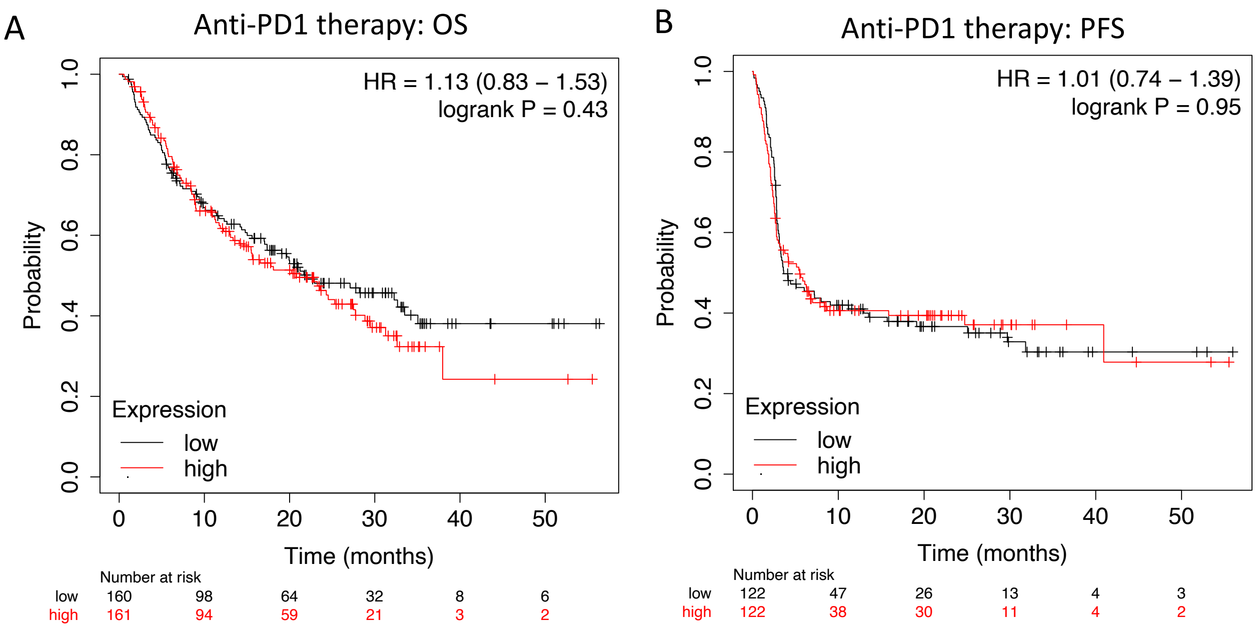

Fig S4. Kaplan-Meier analysis of the 6 genes' expression level in association to patient OS (**C**) and PFS (**D**), using the median of mean expression of the gene set as the cutoff, in a combined cohort of 321 or 244 patients that treated with anti-PD1 therapy [8].
